# Effect of COVID-19 on Kawasaki Disease: Decrease Age of Onset and Increase Skin Manifestation

**DOI:** 10.1186/s12887-021-03060-w

**Published:** 2021-12-13

**Authors:** Hossein Esmaeilzadeh, Negar Mortazavi, Alireza Salehi, Hossein Fatemian, Seyed Mohsen Dehghani, Mohebat Vali, Hossein Molavi Vardanjani

**Affiliations:** 1 Allergy Research Center, Shiraz University of Medical Sciences, Shiraz, Iran; 2 Department of Allergy and Clinical Immunology, Namazi Hospital, Shiraz University of Medical Sciences, Shiraz, Iran; 3 MD/MPH Program, School of Medicine, Shiraz University of Medical Sciences, Shiraz, Iran; 4 Department of Clinical Pharmacy, School of Pharmacy, Shiraz University of Medical Sciences, Shiraz, Iran; 5 Research Center for Traditional Medicine and History of Medicine, Shiraz University of Medical Sciences, Shiraz, Iran; 6 Gastroenterohepatology Research Center, Shiraz University of Medical Sciences, Shiraz, Iran; 7 Student Research Committee, Shiraz University of Medical Sciences, Shiraz, Iran

**Keywords:** Age, COVID-19, Kawasaki, Pandemic, Skin

## Abstract

**Background:**

Kawasaki Disease (KD) is the most common childhood vasculitis and cause of acquired heart disease for no apparent reason. There is some evidence indicating infectious agents as possible triggers for KD. During the COVID-19 pandemic, vasculitis has been a presentation of COVID-19 in children. We performed this study to assess the association between KD and COVID-19.

We evaluated KD hospitalized children during February to September 2020 for COVID-19 (group one) and compared their demographic, clinical, laboratory, and echocardiographic findings with KD patients from the same period time in 2019 (group two). We also compared the same data in COVID-19 positive and COVID-19 negative KD patients in 2020 pandemic period in Shiraz Namazi referral hospital at southwest of Iran.

**Results:**

Thirty-two patients in group one compared with 44 patients in group two. Sixty-eight percent of group one KD patients were positive for COVID-19 during the pandemic period. KD Age of onset in the group one was lower than group two (4.38 years VS 5.5 years, P-value = 0.044). There was no difference in the demographic, clinical, laboratory, and echocardiographic features of the patients during and before the COVID-19 pandemic (p-value > 0.05). Moreover, Comparing COVID-19 positive and negative the incidence of rash was higher within COVID-19 positive cases (p < 0.05), and coronary artery abnormalities were more prevalent in COVID-19 negative cases (p < 0.05).

**Conclusion:**

Admission rate of KD was almost similar during the COVID-19 pandemic but 68% of KD admitted patient were COVID-19 positive. Age of onset for KD during the COVID-19 pandemic was lower and skin manifestation was higher than the same period time in last year.

## Background

Kawasaki Disease (KD) is the most common form of vasculitis in childhood that predominantly affects children under 5 years of age. KD is an acute onset systemic vasculitis involving medium and small-sized arteries with a predilection toward the coronary arteries [1]. Cardiac manifestations in KD include coronary aneurysm/dilatation, aortic root dilatation, myocarditis, and rarely cardiogenic shock. KD is considered to be the most common cause of acquired heart disease in children in developed countries. The certain etiology of KD has not been determined yet, but viruses have been suspected to be a causal factor [2].

The Coronavirus disease 2019 (COVID-19) pandemic caused by a novel mutant coronavirus, also known as severe acute respiratory syndrome coronavirus 2 (SARS-CoV-2), rapidly spread throughout China, and later on throughout the world, resulting in a global health crisis affecting all ages. SARS-CoV-2 infection is usually responsible for mild to moderate respiratory symptoms in children. While COVID-19 appears to be less severe in children, there is a growing concern that the disease might present as post infection immune dysregulation, especially in young asymptomatic children. Post-viral immunological reactions are thought to play an important role in the pathogenesis of this phenomenon. This condition is called “pediatric inflammatory multisystem syndrome temporally associated with SARS-CoV-2 infection (PIMS-TS)” or multisystem inflammatory syndrome in children (MIS-C) that is hard to distinguish from KD [3–6].

An increase in the incidence of KD and alterations in its clinical features have been observed during the COVID-19 pandemic. With the limited number of studies in this regard, and the existence of controversies among scientific societies being into consideration, the present study conducted to investigate the association between KD and COVID-19 and compare with same period of time before COVID-19 pandemic.

## Methods

### Study Design

This retrospective observational study conducted in Namazee tertiary referral center, southwest of Iran (Shiraz, Iran). Thirty-two children with established KD diagnosis during the pandemic of COVID-19 (group one) were studied from February to September 2020 and were compared with medical records of 44 patients with the same diagnosis in the same period in 2019 (group two).

### Inclusion and Exclusion Criteria

The diagnoses of KD was made using American Heart Association diagnostic criteria [7] fever for at least five days together with at least 4 of the 5 following principal clinical features: 1. Erythema and cracking of lips, strawberry tongue, and/or erythema of oral and pharyngeal mucosa 2. Bilateral bulbar conjunctival injection without exudate 3.maculopapular rash 4. Erythema or desquamation of hands and feet 5. Cervical lymphadenopathy (≥ 1.5 cm diameter).

A specific questionnaire was completed which included the demographic information, medication history, laboratory findings (ESR, CRP), and echocardiography results of the patients. Group one patients divided into COVID-19 positive and COVID-19 negative subgroups based on WHO criteria based on COVID-19 PCR (polymerase chain reaction) test [8]. Exclusion criteria were age ≥ 18 years old, patients with multisystem inflammatory syndrome, sepsis or bacterial infection, and patients with other forms of vasculitis. Any COVID-19 positive patient with lung involvement, gastrointestinal involvement, thrombocytopenia or lymphopenia were excluded due to suspected MIS-C. Patients with negative PCR but positive antibody titer or history of SARS-COV-2 positive contact were also excluded due to possible CDC criteria MIS-C [6].

### Statistical Analysis

The clinical presentations and laboratory findings of the groups were separately analysed with IBM SPSS Statistics version 26. One sample K-S test used to determine whether the data followed a normal distribution pattern. The Mann–Whitney U test was performed for comparison of quantitative variables in two study groups. The proportion of qualitative variables we applied Chi squared test and Fisher's exact test. $P$ -value of $<.05$ was considered as statistically significant. Assuming the normal approximation, statistical power (%) was estimated for statistical comparisons. The Ethics committee of Shiraz University of Medical Sciences approved this study.

## Results

### Demographic Data and COVID PCR Results

In total, 76 patients with KD were analysed; 32 children with established KD diagnosis during the 2020 pandemic of COVID-19 (group one) were observed and compared with medical records of 44 patients with KD diagnosis at the same period time in 2019 (group two). The mean age of the total 76 participants was 5.03 ± 3.42 years, and the average hospitalization period was 4.47 ± 3.10 days. In group one, 22 patients (68%) were COVID-19 positive, 10 patients (32%) were COVID-19 negative cases.

### Clinical Characteristics comparing groups

There was a significant difference in KD age of onset between two groups (p-value = 0.044), and KD age of onset was lower in group one than in group two. Comparing COVID-19 positive and negative group one patients showed no significant difference. Rash, Oral mucosal involvement and extremity changes were most clinical manifestation in KD patients in both groups between and during COVID-19 pandemic. There was no significant difference in the clinical presentations, laboratory findings, and echocardiographic characteristics of the two groups (Table 1). Comparing COVID-19 positive and negative patients in group one, rash was more prevalent within COVID-19 positive cases (72% vs 30%, p < 0.05). Regarding the echocardiographic findings including any coronary dilation or aneurysm, the patients who tested negative for COVID-19 were more involved with coronary artery abnormalities (40% vs 31.8%, p = 0.04) (Table 2). Laboratory findings in group one showed in Tables 2 and 3.

**Table 1:** Demographics, clinical, and laboratory findings (group 1 vs group 2)

**Characteristics**	**Total (n = 76)**	**Group 1** **(n = 32)**	**Group 2** **(n = 44)**	**P value**	**Estimated Power (%)**
Age	5.03	4.38	5.50	0.044	29
Male%	60% (n = 46)	65% (n = 21)	56% (n = 25)	0.438	8
Hospital course (days)	4.47	5.13	4.00	0.555	30
Conjunctivitis	38 (50%)	14 (43.8%)	24 (52.2%)	0.353	7
Lymphadenopathy	27 (36%)	9 (28.1%)	18 (39.1%)	0.250	11
Oral mucosa or lips changes	44 (58%)	16 (50%)	28 (60%)	0.160	9.1
Rash	47 (62%)	19 (59%)	28 (60.1%)	0.706	3.5
Extremity changes	44 (58%)	19 (59%)	25 (54.3%)	0.824	3.7
Abnormal echocardiography findings (regarding coronary arteries)	33 (43%)	11 (34%)	12 (26%)	0.534	7.4
ESR(mm/h)	62.01	75.79	57.5	0.068	46.1
CRP (mg/L)	55.00	50.39	58.25	0.707	10

**Table 2:** Group one sub groups in COVID-19 pandemic: Demographic, clinical, and laboratory findings (COVID-19 positive vs COVID-19 negative)

**Characteristics**	**Total (n = 32)**	**COVID-19 Positive cases** **(n = 22)**	**COVID-19** **Negative cases (n = 10)**	**P-value**
Age	4.38	4.32	4.50	0.967
Male%	65% (n = 21)	72% (n = 16)	50% (n = 5)	0.725
Hospital course (days)	5.13	5.77	3.70	0.212
Conjunctivitis	14 (44%)	10 (45.5%)	4 (40%)	0.773
Lymphadenopathy	9 (28%)	7 (31.8%)	2 (20%)	0.491
Oral mucosa or lips changes	16 (50%)	11 (50%)	5 (50%)	0.315
Rash	19 (59%)	16 (72%)	3 (30%)	0.023
Extremity changes	19 (59%)	13 (59%)	6 (60%)	0.961
Abnormal echocardiography findings (regarding coronary arteries)	11 (34%)	7 (31.8%)	4 (40%)	0.04
ESR(mm/h)	75.79	71.05	86.23	0.398
CRP (mg/L)	50.39	53.86	43.10	0.269
Elevated D-Dimer	-	5 (22.7%)	-	-
Elevated Ferritin	-	3 (13.6%)	-	-
Elevated LDH	-	3 (13.6%)	-	-
Elevated Fibrinogen	-	2 (9.09%)	-	-
Elevated Procalcitonin	-	2 (9.09%)	-	-
Decreased ALBumin	-	6 (27.2%)	-	-

**Table 3:** Group one sub groups in COVID-19 pandemic vs Group two: laboratory findings (adjusted to age)

**Characteristics**	**Before the pandemic (n = 44)**	**Pandemic** **(n = 32)**		**P-value** **(Before the pandemic vs positive COVID-19)**	**P-value** **(Before the pandemic vs COVID-19 Negative cases)**
		**COVID-19 Positive cases** **(n = 22)**	**COVID-19** **Negative cases (n = 10)**		
**Leukocytosis**	19(43.18)%	10(45.45)%	3(30.00%)	0.862	0.448
**Anemia**	16(36.36%)	7(31.81%)	4(40.00%)	0.716	0.831
**Increased ALT**	10(22.72%)	7(31.81%)	4(40.0%)	0.429	0.264
**Thrombocytosis**	18(40.90%)	8(36.36%)	5(50.0%)	0.724	0.602

## Discussion

In adults, COVID-19 is typically characterised by pneumonia and respiratory symptoms as well as inflammatory cascade hyperactivation [9, 10], whereas in children the respiratory tract seems not to be the only system infected by SARS-CoV-2 infection [11–13]. There is some evidence indicating that COVID-19 might present in children as a post-infection immune dysregulation. this condition is called “pediatric inflammatory multisystem syndrome temporally associated with SARS-CoV-2 infection (PIMS-TS)” or multisystem inflammatory syndrome in children (MIS-C) seems to be a post SARS-COV-2 infection immune reaction. It often appears few weeks after the SARS-COV-2 infection and only a proportion of patients, maybe 1/3 seems to have a positive PCR at diagnosis [14]. Some of those patients have overlapping features with KD. As mentioned earlier, KD is suspected to have correlation with COVID-19 infection since an increase in KD incidence and changes in its clinical features were observed during the COVID-19 pandemic; however, some controversies exist [15].

We performed this study to investigate possible association between KD and COVID-19. Demographic, clinical, laboratory and echocardiographic data of hospitalized children with established KD diagnosis during COVID-19 pandemic (February to September 2020) were compared with characteristics of KD patients at the same period of time in 2019.

Our study demonstrated that 68% of the total KD patients during the COVID-19 pandemic were COVID-19 positive which was significantly higher than previous literature conducted by Lio K et al. and Verdoni L et al. that reported 14%, and 30% of COVID-19 positive in KD patients [15, 16]. This difference might be due to the higher prevalence of COVID-19 in Iran; moreover, it might suggest that ethnicity and genetic factors may contribute. Our results showed that the demographic information, clinical presentations, laboratory findings, and echocardiographic characteristics of our patients were similar to previous literature in KD. The most common clinical presentation of KD was rash (seen in 62% of patients), oral mucosa and lip involvement (58%), and extremity changes (58%). Our findings were similar to the findings of Cheraghali F et al. [17] introducing skin rash (68.6%) and changes of the lip and oropharyngeal mucosa (60.8%) as the most common manifestations of KD, but were slightly different from Pilania R.K. et al. which showed oral cavity and lips’ changes as the most common manifestation (> 95%) of KD, and rash as the second (> 90%). Furthermore, our results showed that neck lymphadenopathy was the least common clinical finding that is compatible with Pilania R.K. et al. [18].

There was an apparent decrease in KD cases in our referral hospital’s admissions during the COVID-19 pandemic in comparison with the same period last year. Lio K et al. reported a similar drop in KD cases in Tokyo, Japan, in contrast with Verdoni L et al. who showed an increase in disease incidence in Bergamo, Italy. Epidemiological clusters of KD occurrence, seasonal changes and a very low risk of recurrent Kawasaki suggest that several factors can be triggers for KD like infectious agents [19]. Consequently, in our study, KD admission rate decrease was assumed to be a reflection of the decrease in other viral and bacterial infective diseases during the COVID-19 pandemic because of the quarantine and improvement of hygiene; therefore, SARS-CoV-2 infection is probably not an indicating factor for prevalence or incidence of KD and needs further investigation.

There was no significant difference between the demographic information, clinical presentations, laboratory findings, and echocardiographic characteristics between the two groups and between COVID-19 Positive and COVID-19 negative patients in the course of the COVID-19 pandemic; however, there was a significant decrease in the mean age of KD presentation comparing to last year. The results were similar to the ones of Verdoni L et al. that found a difference in the mean age of KD patients during and before the COVID-19 pandemic (3·0 vs 7·5 years). In contrast, Lio K et al. demonstrated that there was no significant difference in mean age of KD presentation. Accordingly, such results can suggest that at younger ages, SARS-CoV-2 infection may increase the prevalence of KD.

We only analysed CRP and ESR results of the patients as the predominant laboratory data for KD diagnosis. CRP and ESR results in KD patients during the pandemic (group 1) were compared to last year records of KD patients (group 2). Our results show no significant difference in laboratory data between the two groups. Our results (ESR = 75.79 mm/h in group 1 vs ESR = 57.5 mm/h in group 2 and CRP = 50.39 mg/L in group 1 vs CRP = 58.25 mg/L in group 2) are similar to Verdoni L et al. research that represent no significant change in ESR and CRP (Mean ESR: 72 mm/h, mean CRP: 25 mg/dL) during COVID-19 pandemic.

In European countries, the proportion of KD patients with coronary artery aneurysm reported were similar to that reported in North America, eg, in Ontario, Canada, during 2004–2006, approximately 4% of KD patients were reported to have developed coronary artery aneurysms and 4.6% with aneurysms in Ireland. Slightly higher proportions of KD patients with coronary artery aneurysm reported in recent studies in Northern France (18%) and Northern Italy (24%) [20, 21]. The incidence of coronary artery dilatations and aneurysms in our study was 43% with no significant difference between COVID-19 pandemic time and before (34% in group 1 and 26% in group 2) that is in contrast with Verdoni L, et al. research that represents an increase in cardiac involvement (2 of 19 vs 6 of 10). Incidence of coronary involvement is significantly higher in our research compared to mentioned literature that can be due to referral of our hospital and difference in ethnicity and genetics that need to be investigated.

In our study the incidence of coronary artery abnormalities was higher in COVID-19 negative cases compared to the ones who tested positive (40% vs 31.8%, p < 0.05). This is compatible with Verdoni L et al., in which 67% (4 of 6) of the patients who were tested negative for COVID-19 demonstrated abnormal coronary artery findings, versus 50% (2 of 4) of COVID-19 positive cases.

The limitation of our study is that the present report based on a retrospective study at a single tertiary referral center in Iran’s southwest. Further studies are needed to clarify the actual relationship between COVID-19 and KD. Since the current study focused only on patients with KD, the exact epidemiology and characteristics of PIMS-TS was not analysed and excluded.

## Conclusion

Sixty-eight percent of KD patients were COVID-19 positive during the pandemic of COVID-19 and the age of onset for KD during the COVID-19 pandemic was lower than the same period of time before the pandemic. Skin rash was significantly higher in COVID-19 positive patients, but coronary involvement was more prevalent in COVID-19 negative patients. We did not find increasing in KD admission rate during the COVID-19 pandemic.

## Availability of supporting data

The datasets used and/or analyzed during the current study are available from the corresponding author on reasonable request.

## Abbreviations

KD: Kawasaki Disease
COVID-19: Coronavirus disease of 2019
PIMS-TS: Pediatric inflammatory multisystem syndrome temporally associated with SARS-CoV-2 infection
MIS-C: Multisystem inflammatory syndrome in children
